# Tolerability and Muscle Activity of Core Muscle Exercises in Chronic Low-back Pain

**DOI:** 10.3390/ijerph16193509

**Published:** 2019-09-20

**Authors:** Joaquín Calatayud, Adrian Escriche-Escuder, Carlos Cruz-Montecinos, Lars L. Andersen, Sofía Pérez-Alenda, Ramón Aiguadé, José Casaña

**Affiliations:** 1 Exercise Intervention for Health Research Group (EXINH-RG), Department of Physiotherapy, University of Valencia, 46010 Valencia, Spain; joaquin.calatayud@uv.es; 2 Department of Physiotherapy, University of Valencia, 46010 Valencia, Spain; sofia.perez-alenda@uv.es; 3 Department of Physiotherapy, University of Malaga, 29071 Malaga, Spain; adrianescriche@gmail.com; 4 Laboratory of Clinical Biomechanics, Department of Physical Therapy, Faculty of Medicine, University of Chile, Santiago 8380453, Chile; ccmkine@gmail.com; 5 National Research Centre for the Working Environment, 2100 Copenhagen, Denmark; lla@nrcwe.dk; 6 Sport Sciences, Department of Health Science and Technology, Aalborg University, 9100 Aalborg, Denmark; 7 Haemostasis and Thrombosis Unit, Universitary and Polytechnic Hospital La Fe, 46026 Valencia, Spain; 8 Department of Nursing and Physiotherapy, University of Lleida, 25003 Lleida, Spain; raiguade@aiguade.com

**Keywords:** bridging, electromyography, plank, squat, trunk

## Abstract

Most of the studies evaluating core muscle activity during exercises have been conducted with healthy participants. The objective of this study was to compare core muscle activity and tolerability of a variety of dynamic and isometric exercises in patients with non-specific low back pain (NSLBP). 13 outpatients (average age 52 years; all with standing or walking work in their current or latest job) performed 3 consecutive repetitions at 15-repetition maximum during different exercises in random order. Surface electromyography was recorded for the rectus abdominis; external oblique and lumbar erector spinae. Patients rated tolerability of each exercise on a 5-point scale. The front plank with brace; front plank and modified curl-up can be considered the most effective exercises in activating the rectus abdominis; with a median normalized EMG (nEMG) value of 48% (34–61%), 46% (26–61%) and 50% (28–65%), respectively. The front plank with brace can be considered the most effective exercise in activating the external oblique; with a nEMG of 77% (60–97%). The squat and bird-dog exercises are especially effective in activing the lumbar erector spinae; with nEMG of 40% (24–87%) and 29% (27–46%), respectively. All the exercises were well tolerated; except for the lateral plank that was mostly non-tolerated. In conclusion; the present study provides a variety of dynamic and isometric exercises; where muscle activity values and tolerability can be used as guide to design evidence-based exercise programs for outpatients with NSCLBP.

## 1. Introduction

Low back pain (LBP) is a major public health challenge and a socioeconomic burden worldwide [1]. LBP is the leading cause of disability in people under 45, generating the greatest economic health-expenditure in the population between 20 and 50 [2] and increasing the risk of long-term sickness absence from work [3,4]. In most cases, people recover spontaneously from the pain regardless of the treatment, however many suffer one or more episodes of recurrence. Pain can be disabling and persist in about 20–30% of cases, limiting activity and functional capacity and deteriorating the quality of life [2]. Part of this persistent and disabling LBP constitutes the non-specific chronic low back pain (NSCLBP), described as pain in the area between the last rib and the gluteal folds that persists for at least 12 weeks without obvious radiological signs of abnormality or another known possible cause [5,6,7]. 

Active exercise for low-back pain is one of the most prescribed treatments [8]. Particularly, specific training of the low back and abdominal muscles or “core stability training” have become a popular treatment [9]. Core stability training involves activating deep and superficial spinal muscles [10]. Exercises providing proper activity (neural drive) have been recommended to train core muscles in NSCLBP [11]. However, most of the studies evaluating core muscle activity during different exercises have been conducted with healthy people and thus exercise selection in rehabilitative programs has been usually based in these data [12]. This limits proper design of therapeutic programs, because patients with NSCLBP have shown altered core activity patterns compared to healthy peers [13,14]. 

To target the core muscles, both isometric and dynamic exercises can be used. However, most of the exercise evaluation studies with electromyography (EMG) in NSCLBP patients have used isometric contractions, underestimating the potential benefits and specific adaptations of dynamic exercises. Moreover, in case of using dynamic exercises in patients with NSCLBP, absolute loads were used, making individualization with a relative intensity for each patient difficult [5,15]. While some isometric exercises provide adequate resistance to enhance core stiffness [16], dynamic exercises provide less angle-specific adaptations, greater dynamic muscle strength gains [17] and can be easily manipulated to gradually increase intensity as the phases of rehabilitation progress. This fact facilitates dose-individualization and may be especially relevant in advanced rehabilitative stages where progressive neuromuscular challenge must be achieved to further enhance adaptations. Furthermore, dynamic exercises are more related to daily life activities and have shown promising core EMG activity, at least in healthy subjects [18]. For instance, a previous study found that the squat exercise generally provided greater longissimus and multifidus activity than other typical exercises performed with a stability ball in healthy subjects [18]. However, studies evaluating core EMG values during different dynamic and isometric exercises in patients with NSCLBP are scarce. 

In addition, patient tolerability of these exercises (together with the EMG values) could provide further insight for a proper exercise selection in therapeutic programs. However, no previous studies have provided such knowledge. Thus, the purpose of this cross-sectional study was to evaluate core muscle activity and tolerability during a variety of exercises (including the most used isometric and other promising dynamic exercises) in patients with NSCLBP, in an effort to help in clinical decision-making. We hypothesized that the brace front plank, the torso-twist, and the squat would provide the highest rectus abdominis, external oblique and erector spinae activity, respectively.

## 2. Material and Methods

### 2.1. Subjects

Outpatients 18 years or older, diagnosed with non-specific chronic low back pain and visiting a local hospital during the year of 2017 were considered candidates for the present study and were asked to participate. Inclusion criteria was as follows: non-specific low back pain present for at least the last 3 months, age between 18–75 years old, being referred to hospital rehabilitation by a physician, having the capacity of understanding the exercises. Participants were excluded if they had undergone spine surgery, if they were taking any medication at the moment the study was conducted or if they had any medical condition in which exercise was contraindicated. A total of 13 patients with NSLBP (4 men and 9 women) voluntarily participated in the study, which was performed during November 2017. All participants were informed about the purpose and content of the investigation. Written informed consent was obtained from all individual participants included in the study. The study conformed to The Declaration of Helsinki and was approved by the Local Ethical Committee (H1496152714192).

### 2.2. Procedures

All the participants had 3 familiarization sessions, with 48h rest between sessions. During the first familiarization session, height (IP0955, Invicta Plastics Limited, Leicester, England) and body mass (Tanita model BF- 350, Tokyo, Japan) were obtained. During the last familiarization session, elastic resistance used during each dynamic exercise was measured for the subsequent experimental measurements. In addition, subjects replied to the following question: *“How would you generally describe your physical activity in your main job?”* participants replied on a 4-point scale: (1) Seated work, (2) Standing or walking work that is not strenuous, (3) Standing or walking work with lifting tasks, (4) Heavy and fast strenuous work.

After the third familiarization session, participants had 48h of rest from the specific exercises before starting the experimental session.

Before the experimental session, several restrictions were imposed on the volunteers: no food, drinks, or stimulants (e.g., caffeine) to be consumed two hours before the sessions and no physical activity more intense than daily activities 24 h before the exercises. All measurements were made by the same two investigators and were conducted in the same facility (a primary care center). This article adheres to the STROBE guidelines [19]. 

Before starting with the EMG protocol, an 11-point numerical rating scale, where 0 = “no pain” and 10 = “the worst possible pain”, was used to assess subject’s perception of LBP intensity during the last week. After that, the EMG protocol started with the preparation of subjects’ skin, followed by electrode placement, maximum voluntary isometric contraction (MVIC) collection and exercise performance. Hair was removed from the skin overlying the muscles of interest and the skin was then cleaned by rubbing with cotton wool dipped in alcohol for the subsequent electrode placement. Electrodes were placed according to established recommendations [20] on the rectus abdominis, external oblique and lumbar erector spinae, on the dominant side of the body. Specifically: electrodes for the rectus abdominis were located 2 cm lateral and across from the umbilicus over the muscle belly; electrodes for the external oblique were placed lateral to the rectus abdominis and directly above the anterior superior iliac spine, halfway between the crest and the ribs at a slightly oblique angle; electrodes for the lumbar erector spinae were placed 2 cm from the spine over the muscle mass, with the iliac crest used to determine the L-3 vertebra.

Pre-gelled bipolar silver/silver chloride surface electrodes (Blue Sensor M-00-S, Medicotest, Olstykke, Denmark) were placed with an inter-electrode distance of 2 cm. The reference electrode was placed between the active electrodes, approximately 10 cm away from each muscle, according to the manufacturer’s specifications. All signals were acquired at a sampling frequency of 1 kHz, amplified and converted from analog to digital. To acquire the surface EMG signals produced during exercise, an ME6000P8 (Mega Electronics, Ltd., Kuopio, Finland) biosignal conditioner was used. All records of myoelectrical activity (in microvolts) were stored on a hard drive for later analysis. Prior to the exercise performance described below, two MVIC of 5 secs were performed for each muscle and the trial with the highest EMG was selected. Participants performed a non-maximal practice trial to ensure that they understood the task. They were asked to exert progressive contraction during 2 s and 3 s of maximal contraction without reaching a pain intensity greater than 4 of 10. Verbal encouragement was provided to motivate all participants to achieve maximal muscle activity. MVICs were based on standardized muscle testing procedures [21] for the (1) rectus abdominis, (2) external oblique and (3) lumbar erector spinae. Specifically, (1) curling up at 40 degrees with arms on the chest and pressing against the resistance with the participant lying on the exercise mat and feet flat on the floor, (2) curling up at 40 degrees with arms on the chest and pressing against the resistance in an oblique direction with the participant lying on the exercise mat, with the feet flat on the floor and the knees bent at 90 degrees, and (3) trunk extension with the participant lying on a bench and pelvis fixated, the trunk was extended against the resistance.

**Figure 1:**
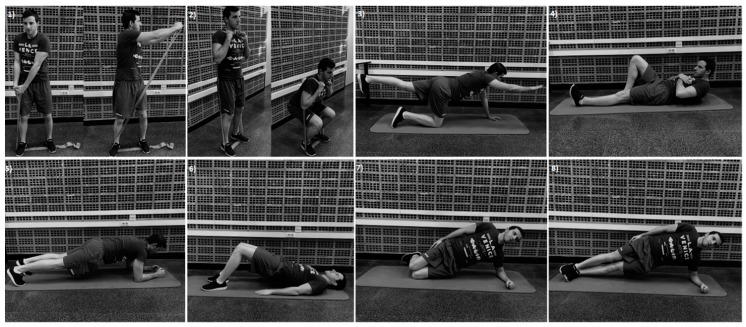
Exercises performed: (**1**) Torso-twist, (**2**) Squat, (**3**) Bird-dog, (**4**) Modified curl-up, (**5**) Front plank/front plank with brace, (**6**) Supine plank, (**7**) Lateral knee plank and (**8**) Lateral plank.

Each subject performed the following nine different exercises (Figure 1), randomly assigned and with 1-min rest interval: torso-twist (from a standing position with feet shoulder-width apart and the elastic band attached to the left feet, with arms positioned horizontally and extended, the patients had to twist their torso from left to right while maintaining feet, legs, and hip stationary); squat (from standing position with feet shoulder-width apart, with the band underneath the feet and the other extreme over the shoulders with both hands gripping the corresponding loop, patients were asked to squat until 90° of knee flexion); bird-dog (from a quadruped position, lifting a leg and the contralateral arm so that the hip and knee were fully extended and the shoulder flexed); modified curl-up (lying with the back on the floor, with one knee flexed and hands underneath the low back, the patients have to slowly raise the chest, shoulders and head); front plank (prone position with the elbows flexed to 90° and knees fully extended, only with the forearms and toes in contact with the ground); front plank with brace (as the previous exercise but with a bracing maneuver, voluntarily contracting the abdominal muscles); supine plank (knees flexed at 90°, both feet resting on the mat and the pelvis lifted and aligned with the thigh), lateral knee plank (in a side-lying position with the dominant side beneath and knees support, with the elbow beneath the shoulder, the other arm perpendicular to the ground, and the pelvis raised); lateral plank (as the previous exercise but without knees support). 

Exercise intensity during the two dynamic exercises (i.e., torso-twist and squat) was 15 repetition-maximum (RM), which was calculated during the last familiarization session. In order to do so, the elastic bands were pre-stretched to approx. 50% of the initial length (initial length, 1.9 m) and then different bands were added when needed to reach the desirable intensity. With this purpose, red, blue, black, silver and gold elastic band colors were allowed (TheraBand CLX, The Hygenic Corporation, Akron, OH, USA), alone or in combination. During the performance of the dynamic exercises, they were asked to use minimal lower body and trunk movement and to perform the exercises without sudden jerks or accelerations for 3 consecutive repetitions. A metronome was used to standardize movement velocity at 1.5-s rate for concentric and 1.5-s rate for eccentric. In regard of the isometric exercises, patients had 2–3 s to reach the proper exercise position, and then they have to maintain it during 5 s, where EMG signal was recorded. Verbal feedback was provided to start or end the exercise and to maintain the proper position. A trial was discarded and repeated if participants were unable to perform the exercise properly.

After each condition, participants were asked to rate tolerability of each individual exercise, according to the following 5-point scale: very tolerated, tolerated, neutral, little tolerated, not tolerated.

A priori power analysis conducted in G*Power (3.1.9.2 version) software (Heinrich-Heine-Universität Düsseldorf, Düsseldorf, Germany) showed that 13 subjects in this design were sufficient to obtain a statistical power of 0.80 at a medium effect size (f = 0.30), with an alpha = 0.05.

### 2.3. Data Analysis

EMG data processing was performed using custom-made algorithms implemented in MATLAB (The MathWorks, Inc., Natick, Massachusetts, USA, version R2015a) software. During later analysis, all raw EMG signals obtained during the exercises were digitally filtered, consisting of (1) high-pass filtering at 10 Hz and (2) a moving root-mean square (RMS) filter of 500 ms. The RMS routine was performed using a 500-ms smoothing filter/window across the entire signal (i.e., across all contractions). The RMS filter was centered by performing a root mean-square routine 250 ms backwards and 250 ms forward from each data point. During the dynamic exercises, for each individual muscle, peak RMS EMG of each of the three repetitions performed was determined, and the average value of these three repetitions was then normalized to the maximal RMS EMG obtained during the experimental session (maximum maximorum EMG of each muscle) [22]. For the isometric exercises, for each individual muscle, the three highest peaks RMS EMG were averaged in each exercise and then normalized to the maximal RMS EMG obtained during the experimental session (maximum maximorum EMG of each muscle). 

### 2.4. Statistical Analyses 

Analyses were performed in MATLAB. The normal distribution of peak RMS normalized EMG was tested using Shapiro-Wilks. The Friedman analysis was applied to assess normalized EMG (nEMG) differences in each muscle during exercises, as well as to assess tolerability differences. Post *hoc* with Bonferroni correction for multiple comparisons was applied when there were multiple random comparisons. Statistical significance was set to *p* < 0.05. nEMG data from the current study were expressed as the median and interquartile range 1 and 3.

## 3. Results

The demographic characteristics of the participants of the current study are the following: age, 52 (7 SD) years; height, 163.8 (7.8 SD) cm; body mass, 77.8 (24.4 SD) kg and low back pain intensity, 4.3 (2.9 SD). All the subjects considered their job as “standing or walking work that is not strenuous”.

**Figure 2:**
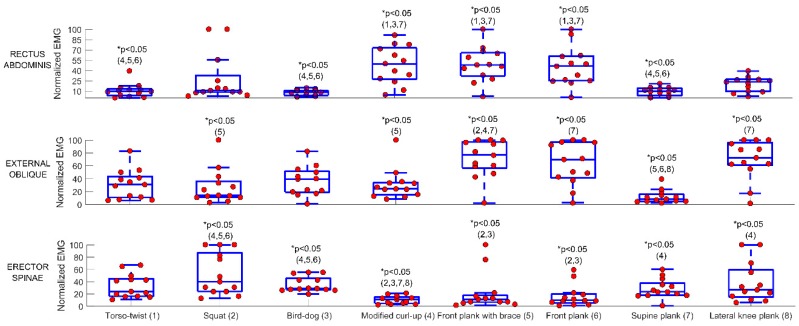
EMG during 8 of the 9 exercises (i.e., the lateral plank was not included due to its general non-tolerability). Circles show individual data. Numbers between parentheses denote statistical differences between the exercises (e.g., number 1 means different from the exercise number 1, which is the torso-twist).

Complete nEMG data induced by the different exercises are presented in Figure 2. The front plank with brace, front plank and modified curl-up caused the greatest number of between-exercise statistical differences for the rectus abdominis, without difference between these, and a median nEMG of 48% (34–61%), 46% (26–61%) and 50% (28–65%), respectively. The front plank with brace induced the greatest number of between-exercise statistical differences for the external oblique, with a nEMG of 77% (60–97%). The squat and bird-dog exercises provided the greatest number of between-exercise statistical differences for the erector spinae, with nEMG of 40% (24–87%) and 29% (27–46%), respectively. *P*-values of post-hoc comparison between muscle activity of each exercise in the individual muscles are reported in Table 1.

**Figure 3:**
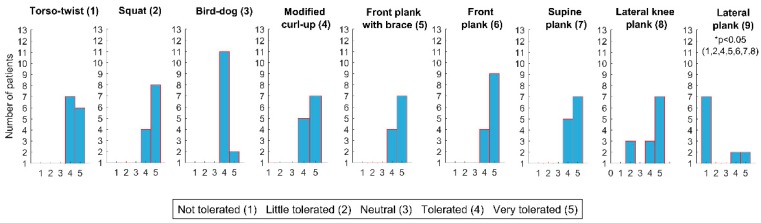
The tolerability of each exercise.

All exercises were very tolerated except the lateral plank that was not tolerated in general and the front plank with brace that was generally only tolerated. Tolerability of each exercise is shown in Figure 3. *P*-values of post-hoc comparison between tolerability of each exercise are shown in Table 2.

## 4. Discussion

The main findings of the study are (1) with the exception of the lateral plank, 8 out of 9 exercises were well tolerated, and (2) the effectiveness of both front plank exercises (with and without brace) and the modified curl-up for activating the rectus abdominis, the effectiveness of the front plank with brace for activating the external oblique and the effectiveness of the squat and bird-dog exercises for activing the lumbar erector spinae.

Most of the previous studies evaluating a battery of core stability exercises with the aim of providing evidence-based clinical guidelines were based on EMG in healthy people [12,23]. Recent examples are two studies [12,24]. evaluating a variety of plank variations performed with and without suspension in healthy university students. These previous works in healthy people also found that the front plank was effective in providing activity for the rectus abdominis and the external oblique. In accordance, we found that this exercise (with and without brace) in NSCLBP patients provides greater EMG values for the external oblique than for the rectus abdominis. It is worth mentioning that the brace maneuver did not result in additional rectus abdominis activity during the front plank, which supports previous findings in NSCLBP patients performing isometric push-ups with/without brace [25]. Together with both front planks, the modified curl-up was the other exercise achieving the greatest number of rectus abdominis activity differences between exercises. In agreement with our findings, other studies reported that the modified curl-up mainly activates the rectus abdominis while provides less external oblique activity [26]. 

An interesting finding in our study was the moderate external oblique activity yielded by the torso-twist exercise, which was considered a promising option for patients with LBP based on data with healthy sample [27]. Our EMG results show that this exercise is not superior than any other exercise. In this sense, the greatest number of between-exercises differences in external oblique activity was provided by the front plank with brace. This contrast is a good example of the importance of performing such exercise evaluation studies in the specific patient-group of interest, instead of solely relying on results from healthy subjects. In healthy participants, the lateral plank exercise has been considered ideal to activate the quadratus lumborum and the abdominal muscles while generates minimum spinal loading [28]. However, it is worth to mention that in our study, only 3 patients were able to perform the regular lateral plank (i.e., without knee support) and thus we did not add EMG data from this exercise. This general absence of tolerability reported by patients with NSCLBP has relevant practical applications, suggesting that the regular version should be considered an advanced exercise and only should be used when patients have proper physical conditioning and adequate technique. 

Partly, supporting the hypothesis of the present study, it was found that the squat, together with the bird-dog, were especially effective in activing the lumbar erector spinae. However, these exercises only induced greater activity than the modified curl-up and both front planks. Partly in accordance, other studies in NSCLBP patients [15,29] found that the isometric squat performed without external loads and the lateral plank provided the highest activity of the erector spinae and moderate activity of the rectus abdominis and external oblique. The fact that external loads can be added during the squat make this exercise interesting and easy to adapt to different patients, whereas manipulation to increase relative intensity for each patient during isometric planks is more limited. In addition, the squat exercise can provide additional lower-limb and functional benefits, since replicates one of the most typical daily activities. This can be especially relevant for those NSCLBP in more advanced age, were moving from a sitting position to a standing position is crucial for maintaining independence [30]. 

Our results suggest that greater erector spinae activity may have been achieved by using higher intensities, although we do not know whether this would have resulted in tolerability problems. Interestingly, other exercises designed to challenge low back muscles as the supine plank have shown relatively low EMG values in healthy participants (even when the exercise was performed with suspension training) [24] or in NSCLBP patients with the exercise performed under instability with knees fully extended [31]. 

A novel aspect of the study was to measure exercise tolerability, which should be used together with the EMG results for a more proper decision-making (e.g., reducing risk of increasing pain) and to improve exercise adherence. As could be expected, the highest rates of tolerability were achieved during the supine plank, which could be considered an easy-to-perform exercise, as also corroborated by the EMG data provided. Besides the lateral plank, which was mostly non-tolerated, other exercises that should be more carefully prescribed and supervised in NSCLBP patients are the lateral knee plank and the modified curl-up, probably due to a difficulty in some patients to maintain the exercise position and a possible transitory pain exacerbation at the shoulder or the neck respectively. However, all the exercises with the exception of the lateral plank were generally very tolerated and could be performed without problems after familiarization. The general non-tolerability showed by the lateral plank has relevant clinical implications and suggest that before using this exercise, patients should start with the knee-supported version in order to progressively improve physical conditioning without excessive physical stress. 

The main limitation of the current study could be the small sample size. However, an a priori power analysis showed that our selected sample size was sufficient. Future studies should corroborate these findings with a larger sample size, measuring deep muscles if possible and evaluating the association between nEMG and pain or other biopsychosocial measures (e.g., kinesiophobia). In addition, well-designed randomized controlled trials are needed to evaluate the efficacy of these exercises on physical function, pain and quality of life in NSCLBP patients.

## 5. Conclusions

With exception of the lateral plank, 8 out of the 9 exercises can be implemented in NSCLBP participants to progress in muscle activity. Specialists must take into account the non-tolerability provided by the lateral plank and should consider the knee variation as the first option of this exercise until proper physical conditioning and exercise technique will be achieved. 

The front plank with brace, front plank and modified curl-up can be considered the most effective exercises in activating the rectus abdominis. The front plank with brace can be considered the most effective exercise in activating the external oblique, whereas the squat and bird-dog exercises are especially effective in activing the lumbar erector spinae. Specialists can choose from a variety of dynamic and isometric exercises, where muscle activity values and tolerability can be used as guide to design evidence-based exercise programs for outpatients with NSCLBP.

## Figures and Tables

**Table 1:** *p*-Values of post-hoc comparison between muscle activity of each exercise in the individual muscles.

Exercise	Exercise	RECTUS ABDOMINIS	EXTERNAL OBLIQUE	ERECTOR SPINAE
1	2	1.000	1.000	1.000
1	3	1.000	1.000	1.000
1	4	**0.004**	1.000	0.066
1	5	**0.001**	0.111	1.000
1	6	**0.014**	0.567	0.699
1	7	1.000	0.291	1.000
1	8	0.696	0.457	1.000
2	3	1.000	1.000	1.000
2	4	0.180	1.000	0.000
2	5	0.074	**0.029**	**0.009**
2	6	0.454	0.182	**0.003**
2	7	1.000	0.858	1.000
2	8	1.000	0.142	1.000
3	4	**0.001**	1.000	**<0.001**
3	5	**<0.001**	0.111	**0.029**
3	6	**0.002**	0.567	**0.012**
3	7	1.000	0.291	1.000
3	8	0.180	0.457	1.000
4	5	1.000	**0.022**	1.000
4	6	1.000	0.142	1.000
4	7	**0.002**	1.000	**0.029**
4	8	1.000	0.111	**0.006**
5	6	1.000	1.000	1.000
5	7	**0.001**	**<0.001**	0.699
5	8	1.000	1.000	0.231
6	7	**0.009**	**<0.001**	0.366
6	8	1.000	1.000	0.111
7	8	0.506	**<0.001**	1.000

Exercises number: (1) Torso-twist, (2) Squat, (3) Bird-dog, (4) Modified curl-up, (5) Front plank with brace, (6) Front plank, (7) Supine plank, (8) Lateral knee plank. Bold numbers indicate a statistical difference (*p* < 0.05).

**Table 2:** *p*-values of post-hoc comparison between tolerability of each exercise performed.

Exercise	Exercise	*p*-Value
1	2	1
1	3	1
1	4	1
1	5	1
1	6	1
1	7	1
1	8	1
1	9	**0.002**
2	3	1
2	4	1
2	5	1
2	6	1
2	7	1
2	8	1
2	9	**<0.001**
3	4	1
3	5	1
3	6	0.360
3	7	1
3	8	1
3	9	0.472
4	5	1
4	6	1
4	7	1
4	8	1
4	9	**0.001**
5	6	1
5	7	1
5	8	1
5	9	**0.012**
6	7	1
6	8	1
6	9	**<0.001**
7	8	1
7	9	**0.001**
8	9	**0.009**

Exercises number: (1) Torso-twist, (2) Squat, (3) Bird-dog, (4) Modified curl-up, (5) Front plank with brace, (6) Front plank, (7) Supine plank, (8) Lateral knee plank. Bold numbers indicate a statistical difference (*p* < 0.05).
